# Stroke or Seizure? Diagnostic Role of Neuroimaging in Acute Neurologic Mimics

**DOI:** 10.3390/neurosci7030071

**Published:** 2026-06-15

**Authors:** Federico Tosto, Concetta Lobianco, Giuseppe Magro, Angelo Pascarella

**Affiliations:** 1 Department of Neuroscience, “Giovanni Paolo II” Hospital, 88046 Lamezia Terme, Italy; tostofederico@hotmail.it (F.T.); giuseppemagro.neuro@gmail.com (G.M.); 2 Unit of Neurology, Sant’Elia Hospital, 93100 Caltanissetta, Italy; clobianco@virgilio.it; 3 Department of Medical and Surgical Sciences, Magna Graecia University, 88100 Catanzaro, Italy; 4 Regional Epilepsy Centre, Great Metropolitan “Bianchi-Melacrino-Morelli Hospital”, 89124 Reggio Calabria, Italy

**Keywords:** acute ischemic stroke, arterial spin labeling, brain CT, brain MRI, DWI, perfusion, stroke mimics, status epilepticus

## Abstract

Background: Acute focal neurological deficits require rapid differentiation between ischemic stroke and stroke mimics to avoid treatment delays and inappropriate therapy. Seizures, including ictal deficits, status epilepticus, and post-ictal/Todd’s phenomena, are among the most challenging mimics. This review summarizes the role of multimodal neuroimaging in distinguishing acute ischemic stroke from seizure-related deficits. Methods: We performed a focused narrative review of neuroimaging findings in acute stroke mimics, emphasizing non-contrast computed tomography (CT), CT angiography, CT perfusion, magnetic resonance imaging (MRI), including diffusion weighted imaging (DWI), apparent diffusion coefficient (ADC), fluid attenuated inversion recovery (FLAIR), and arterial spin labeling (ASL) sequences. Imaging patterns, diagnostic pitfalls, and practical clues for hyperacute stroke pathways were synthesized. Results: Acute ischemic stroke is typically suggested by vascular-territorial abnormalities, including arterial occlusion or stenosis, territorial hypoperfusion, and congruent DWI/ADC restriction. Seizure-related deficits more often show non-territorial cortical perfusion changes, ictal or status-related hyperperfusion, reversible MRI abnormalities, and absence of arterial occlusion. However, post-ictal hypoperfusion, peri-ictal diffusion restriction, and reperfusion-related hyperperfusion may overlap with ischemic patterns. Conclusions: A multimodal approach integrating vascular imaging, perfusion distribution, DWI/ADC, ASL, clinical timing, and EEG findings can improve diagnostic accuracy in the stroke–seizure differential without delaying treatment in true acute ischemic stroke.

## 1. Introduction

Stroke mimics represent a significant challenge in acute neurological care, accounting for nearly one-third of cases initially suspected to be acute ischemic events [1]. While epileptic seizures are the most frequent mimics, other important causes include migraine, functional neurological disorders, brain tumors, infections, and toxic-metabolic encephalopathies [1,2,3,4]. Within this diagnostic spectrum, the clinical differentiation between acute ischemic stroke (AIS) and seizure-related phenomena remains a challenge in emergency neurology [5]. In the hyperacute phase, these two entities often present with overlapping clinical features, including focal neurological deficits, impaired consciousness and speech disturbances [1]. This clinical overlap becomes particularly problematic when seizures are unwitnessed, when post-ictal deficits such as Todd’s paralysis dominate the presentation, or when non-convulsive status epilepticus (SE) produces focal signs without overt motor activity [6]. In these time-dependent scenarios, there is a critical necessity to rapidly identify AIS, particularly large-vessel occlusions and salvageable tissue that may benefit from reperfusion therapy [7]. While misidentifying a stroke as a post-ictal state leads to missed therapeutic windows, unnecessary thrombolysis in mimics, though generally safe, complicates clinical pathways and delays appropriate treatment for the underlying cause [8].

While EEG remains the cornerstone for the diagnosis of seizure-related deficits, neuroimaging is central in confirming ischemia or recognizing patterns that argue against a purely vascular mechanism. Beyond the standard non-contrast computed tomography (NCCT) and CT angiography (CTA), which represent the backbone of initial stroke evaluation, advanced modalities such as CT perfusion (CTP) and magnetic resonance imaging (MRI), including diffusion-weighted imaging (DWI), apparent diffusion coefficient (ADC) maps and Arterial Spin Labeling (ASL), provide critical insights into cerebral hemodynamics and tissue viability [9,10]. At the same time, both CTP and brain MRI offer useful clues in seizure-related evaluations [11,12], frequently revealing hyperperfusion patterns. Despite their utility, these modalities may present significant pitfalls, including “DWI-negative” strokes and seizure-induced perfusion changes that can mimic ischemic signatures [10,13].

This narrative review aims to synthesize the current evidence regarding the diagnostic value of various neuroimaging modalities in distinguishing stroke from seizure, providing a framework for optimized decision-making in the emergency department.

## 2. Materials and Methods

For this narrative review, a comprehensive literature search was performed across PubMed/MEDLINE and Google Scholar for relevant studies published up to March 2026. A combination of Medical Subject Headings (MeSH) terms and free-text keywords related to stroke, seizure and neuroimaging was employed. In detail, the search terms included “stroke”, “seizure”, “status epilepticus”, “stroke mimics”, “neuroimaging”, “brain computed tomography”, “brain magnetic resonance imaging”, “CT angiography”, “CT perfusion”, “arterial spin labeling”. No chronological restrictions were applied. Only articles published in English were included. We considered randomized controlled trials, observational studies, case reports, systematic reviews, meta-analyses and mechanistic experimental research. Furthermore, the reference lists of retrieved articles were manually screened to ensure a thorough selection of pertinent studies. The final bibliography included original research and key reviews, selected based on their quality, originality, and direct relevance to the purpose of this review. Literature quality was assessed based on methodological soundness; prioritization was given to prospective or retrospective cohort studies and clinical trials that employed a clear reference standard and provided detailed imaging parameters. Case reports and small case series were excluded unless they described novel or atypical neuroimaging findings of critical value. Studies were deemed directly relevant if they evaluated the performance of acute neuroimaging (multi-modal CT and MRI) in differentiating stroke from seizure-related mimics or provided information on acute stroke or peri-ictal/post-ictal states imaging abnormalities.

## 3. Results

### 3.1. The Baseline: Non-Contrast CT and CT Angiography

Non-contrast CT is the first-line imaging modality in acute neurological emergencies due to its speed, wide availability, and high sensitivity for intracranial hemorrhage. Its primary role is the exclusion of hemorrhagic stroke and other structural lesions that may contraindicate reperfusion therapy. Early ischemic changes on NCCT, such as loss of gray–white matter differentiation, insular ribbon sign, and hyperdense artery sign, can be detected within the first hours but remain relatively insensitive in hyperacute presentations [14,15]. In the context of acute neurologic mimics, NCCT is frequently non-diagnostic. In seizure-related deficits, including post-ictal states and focal SE, NCCT is most often normal, although transient cortical swelling or subtle hypodensities may occasionally be observed. These findings are non-specific and may overlap with early ischemic changes, limiting their standalone diagnostic utility [16,17].

CTA has become an essential extension of NCCT in modern stroke pathways, enabling rapid assessment of intracranial and extracranial vessels. The identification of a large vessel occlusion (LVO), particularly in the internal carotid artery (ICA) or proximal middle cerebral artery (MCA), strongly supports an ischemic etiology and directly determines eligibility for mechanical thrombectomy [18]. From a differential diagnostic perspective, CTA plays a key role in identifying stroke mimics. The absence of LVO in patients with severe focal deficits should raise suspicion for alternative diagnoses, particularly seizures, migraine, or functional disorders. Observational studies show that a substantial proportion of suspected stroke patients without vascular occlusion are ultimately diagnosed with mimics, with seizures being among the most frequent causes [19,20]. However, CTA has limitations. Distal occlusions, transient embolic events with spontaneous recanalization, or suboptimal contrast timing may lead to false-negative results. In addition, seizure-related hemodynamic changes may occasionally produce misleading vascular appearances [21]. Therefore, CTA should always be interpreted in conjunction with clinical presentation and subsequent multimodal imaging when diagnostic uncertainty persists.

Overall, NCCT and CTA constitute the baseline imaging framework for acute neurological assessment. While NCCT primarily excludes hemorrhage, CTA provides crucial vascular information that helps distinguish ischemic stroke from mimics. Importantly, the absence of both hemorrhage and LVO in a patient with significant neurological deficit should prompt early consideration of seizure-related pathology and escalation to perfusion imaging or MRI.

### 3.2. CT Perfusion: Beyond the Ischemic Core

CTP complements CTA and is a well-established method for tissue-based selection of reperfusion therapies in acute ischemic stroke within the extended treatment window [22,23,24,25,26,27]. The key perfusion parameters include cerebral blood flow (CBF), cerebral blood volume (CBV) and mean transit time (MTT), which are connected via the central volume principle: CBF = CBV/MTT [22,28,29,30]. In acute ischemic stroke, the earliest abnormality is often increased MTT (i.e., the average time required for blood to traverse a brain region). The ischemic penumbra is characterized by prolonged MTT, reduced CBF and preserved or slightly increased CBV, reflecting residual autoregulatory vasodilation in viable tissue at risk. Conversely, the infarct core shows prolonged MTT with marked reductions in both CBF and CBV, indicating irreversible or near-irreversible ischemic injury [22,23,24,29]. The clinical utility of CTP has been established in various studies. Several randomized trials, including EXTEND-IA and EXTEND, have validated the role of CTP-based selection for reperfusion therapies in acute ischemic stroke, supporting its use in identifying patients with salvageable tissue beyond conventional time windows [26,27]. However, despite its clinical utility, CTP represents a time-specific estimation of cerebral perfusion and does not directly visualize infarction [3,24]. It identifies tissue at risk or already infarcted based on perfusion thresholds, which may vary across software platforms. Consequently, small perfusion defects may be missed, and both ischemic core and penumbra estimates may be influenced by acquisition and post-processing variability [23,24].

Seizure-related abnormalities typically present as cortical hyperperfusion or cortical–subcortical hypoperfusion extending beyond vascular territories and occurring without corresponding vessel occlusion [28]. Once vessel occlusion has been excluded on CTA, CTP becomes particularly useful in differentiating these patterns from ischemic stroke [28,29,30]. While ischemic stroke is defined by territorial deficits with decreased CBF, ongoing seizures or SE typically present with increased or normal CBF and CBV, and a decreased or normal MTT [22,29]. Cortical hyperperfusion has been reported as the most frequent seizure-related pattern (59.5%), followed by cortical-subcortical hypoperfusion (40.5%), with both patterns crossing vascular territories and showing no vessel occlusion on CTA [28]. This is supported by Lucas et al., who found that in a code-stroke cohort without vascular occlusion on CTA, CTP abnormalities were present in 73% of seizures versus 40% of strokes. Hyperperfusion was markedly more frequent in seizures (36% vs. 2%), while hypoperfusion occurred at similar rates (38% in both groups). Non-territorial perfusion patterns were also substantially more common in seizures (44% vs. 2%) [30]. Similarly, Gugger et al. and Strambo et al. identified hyperperfusion as the primary finding in ongoing seizures and SE, often in the absence of ischemic signs [29,31], while Todd’s phenomenon was most often associated with normal perfusion, followed by hypoperfusion and then hyperperfusion in a minority of cases [29]. Furthermore, focal CTP hyperperfusion has been reported to have a sensitivity of 60% and a specificity of 77.8% for SE, with thalamic hyperperfusion demonstrating exceptionally high specificity for the condition [32,33]. Hypoperfusion represents the main diagnostic pitfall of CTP, as it may occur both in ischemic stroke and in seizure, mainly in post-ictal states. However, seizure-related hypoperfusion is typically milder, with relatively preserved CBF and CBV and less pronounced MTT changes and often does not conform to vascular territories [28,31]. In contrast, territorial perfusion abnormalities are strongly associated with ischemic stroke, particularly when supported by corresponding CTA findings. For example, in isolated aphasia, a territorial CTP deficit predicts stroke, whereas non-territorial hypoperfusion or the absence of CTA abnormalities is not associated with infarction on follow-up imaging [34]. This aligns with the low sensitivity but high specificity of multimodal CT in stroke mimics, highlighting the risk of false-positive interpretation of hypoperfusion patterns [35].

Overall, the key discriminator lies in the spatial distribution and vascular correlation of perfusion abnormalities: territorial deficits supported by CTA findings indicate ischemic stroke, whereas CTA-negative, non-territorial abnormalities, especially cortical or thalamic hyperperfusion, suggest seizure [28,29,30,31,32,34,35]. Isolated hypoperfusion alone is insufficient to diagnose infarction, as post-ictal changes may mimic ischemia but are typically less severe and non-territorial [28,31,34,35].

### 3.3. Magnetic Resonance Imaging: The Role of DWI and FLAIR

MRI is more sensitive and specific than CT for the early detection of AIS, although its availability may be limited in the hyperacute setting [10]. Within minutes to hours of arterial occlusion, DWI reveals cytotoxic edema as hyperintense areas with corresponding ADC hypointensity, indicating restricted water diffusion [36]. However, early DWI lesions do not always correspond to the final infarct core, as partial or complete DWI reversal (DWIR) may occur, particularly after early reperfusion therapies, and is associated with better neurological outcomes in both small (<1 mL) and large (≥70 mL) strokes. This phenomenon can occur across a wide range of infarct sizes and may lead to overestimation of irreversible tissue in the hyperacute phase [36]. To improve accuracy, DWI is often paired with perfusion-weighted imaging (PWI), which assesses mean transit time, cerebral blood flow, and cerebral blood volume to identify a mismatch that defines the hypoperfused ischemic penumbra, guides thrombolysis selection, and predicts the final infarct area [37]. FLAIR imaging helps estimate lesion age. A lesion visible on DWI but not yet hyperintense on FLAIR suggests stroke onset within the last 4.5 h, a mismatch validated by the WAKE-UP trial to safely guide intravenous alteplase administration in wake-up strokes or those of unknown onset [37,38]. The WAKE-UP trial also revealed that, beyond time estimation, MRI is also useful in posterior circulation stroke detection, stroke mimics differentiation, and etiological assessment in the subacute phase, where lesion distribution may suggest small-vessel disease, large-artery disease, or cardioembolic sources [36,39]. Indeed, in terms of lesion distribution, watershed infarcts are often associated with stenosis or occlusion of proximal arteries, while multiple DWI lesions involving multiple vascular territories suggest a proximal source of embolism, such as the heart or aorta [31]. Rarely, such multi-territorial patterns can also reflect neoplastic hypercoagulability [40,41]. Despite its utility, DWI interpretation is fraught with potential false negatives and false positives. False-negative initial scans occur in 0% to 33% of AIS patients and are highly associated with shorter times to imaging, lower NIHSS scores at onset, and specific syndromes like ataxic hemiparesis, internuclear ophthalmoplegia, or lateral medullary syndrome [42]. An important meta-analysis of 3236 patients demonstrated that posterior circulation strokes are five times more likely to present with a negative initial DWI compared to anterior circulation strokes [43]. This finding can be explained by technical factors, such as susceptibility artifacts or standard 5 mm slice thicknesses missing small brainstem lesions, suggesting the adoption of thinner 3 mm protocols for high-suspicion cases [44]. Conversely, false positives arise when non-ischemic conditions mimic the cytotoxic edema typical of AIS.

Seizures are a particularly common mimic driven by the first mechanism: neuronal hyperexcitability and increased metabolic demand cause Na/K pump failure, leading to intracellular water accumulation and cellular swelling, appearing on brain MRI as hyperintensity on DWI and reduced ADC. This typically affects mesial temporal structures and the corpus callosum, with an incidence of ictal-related MRI changes varying from 0.007% to 20.6% [12,36]. The manifestation of these changes likely depends on factors like seizure duration and time to imaging [45]. Differentiating them from early cortical stroke relies on identifying a nonvascular distribution, absence of vascular occlusion, and normal or increased perfusion [46,47]. DWIR can occur in both scenarios, but while ischemic reversal localizes to the reperfused penumbra area, epileptic reversal occurs in the cortex and associated network structures; however, SE often yields more variable findings, presenting a mix of cytotoxic, vasogenic, reversible, and irreversible changes due to permanent cellular damage [12,46,47,48].

Ultimately, distinguishing AIS from clinical stroke mimics demands a comprehensive assessment integrating patient history, physical examination, and EEG or laboratory findings, alongside a meticulous evaluation of DWI lesion location, appearance, persistence, and vascular distribution [36].

### 3.4. Arterial Spin Labeling: Unmasking Ictal Hemodynamics

ASL is a non-contrast MRI perfusion technique that estimates CBF using magnetically labeled arterial water as an endogenous tracer, offering the primary advantage of evaluating perfusion in the acute neuroimaging setting without gadolinium administration. While pseudocontinuous ASL is generally preferred in clinical practice, its interpretability depends on technical variables such as labeling efficiency, post-labeling delay, arterial transit time, field strength, motion, and signal-to-noise ratio. In AIS, these limitations are particularly relevant because delayed collateral-dependent flow can produce arterial transit artifacts rather than true tissue perfusion [49].

In AIS, ASL typically demonstrates reduced CBF within a vascular territory corresponding to arterial occlusion or upstream stenosis. While AIS typically presents as reduced CBF congruent with an arterial territory, delayed collateral arrival can generate high intravascular signals at the margins of the perfusion deficit; although this artifact can indirectly depict collateral flow, it also complicates CBF quantification and can lead to an overestimation of hypoperfusion [50]. When combined with DWI, a DWI–ASL mismatch may suggest hypoperfused but not yet infarcted and potentially salvageable tissue; however, prolonged arterial transit times may lead to overestimation of hypoperfusion [51].

The fundamental rationale for using ASL in differential diagnosis between stroke and seizure is that ictal activity increases neuronal metabolism, producing regional, non-territorial cortical hyperperfusion in the epileptogenic zone during the ictal phase [13]. In acute seizure presentations, ASL typically shows lateralized cortical hyperperfusion with good correlation to EEG localization. Additional findings such as ipsilateral thalamic (pulvinar) hyperperfusion and contralateral cerebellar hyperperfusion (diaschisis) may further support an epileptic origin and improve specificity compared with other causes of hyperperfusion, including migraine or post-stroke “luxury perfusion” [13]. Indeed, the differential diagnostic challenge with stroke is further heightened by “luxury perfusion,” or reperfusion following spontaneous or treatment-related recanalization, which manifests as increased parenchymal perfusion in previously ischemic tissue. Because this can closely resemble seizure-related hyperperfusion, ASL cannot be interpreted using a simplistic dichotomy where hypoperfusion equates to stroke and hyperperfusion equates to seizure; accurate differentiation strictly requires considering territoriality, vascular patency, diffusion restriction, and clinical evolution [13].

ASL is especially sensitive in SE, where hyperperfusion is the predominant pattern and reflects sustained ictal activity and increased metabolic demand. Kim et al. studied patients with SE and self-limiting seizures using multi-delay ASL, dynamic susceptibility contrast (DSC), FLAIR, and DWI, finding that ASL detected relevant perfusion abnormalities in 90.2% of SE cases, significantly outperforming other imaging sequences [43]. Thalamic involvement is frequently observed (approximately half of SE cases, often ipsilateral to the cortical abnormality) and supports the concept of network-level propagation. While compared with conventional MRI sequences such as DWI, FLAIR, and DSC perfusion, ASL demonstrates higher sensitivity for detecting SE–related abnormalities, its prognostic value and specificity remain limited. It should therefore be considered a complementary tool rather than a standalone diagnostic method [52]. The post-ictal phase introduces a major diagnostic trap. While ongoing seizures and SE predominantly show hyperperfusion, self-limiting seizures or Todd’s paresis often result in normal perfusion or hypoperfusion [53]. In the Kim et al. cohort, ASL abnormalities were less frequent after self-limiting seizures than SE, and nearly half of those abnormal cases exhibited hypoperfusion [54]. This overlap is clinically relevant because post-ictal hypoperfusion can mimic ischemic stroke, particularly in unwitnessed seizures or when focal neurological deficits are present without clear vascular occlusion. In this context, perfusion direction alone may be insufficient for diagnosis and interpretation must integrate vascular imaging, diffusion findings, timing, clinical evolution, and EEG correlation [54].

Ultimately, practical pattern recognition must integrate three elements: territoriality, direction of perfusion change, and associated network signs. AIS typically produces territorial hypoperfusion respecting an arterial territory and consistent with arterial occlusion, stenosis, or collateral delay, whereas seizure-related changes are cortical, non-territorial, predominantly hyperperfused during the ictal phase, and frequently with pulvinar, thalamic, or crossed cerebellar involvement. These distinctive ASL signs are exceptionally valuable in “code stroke” scenarios where EEG is not immediately available or conventional MRI sequences are equivocal [13,49,50,51,52,53,54,55].

## 4. Clinical Implications and Future Directions

The distinction between acute ischemic stroke and seizure-related focal deficits remains a frequent diagnostic challenge in acute clinical practice. Both conditions may present with sudden focal neurological symptoms and, not uncommonly, with incomplete or unreliable clinical information, including unwitnessed symptom onset. In this context, neuroimaging is often highly informative and frequently decisive in clinical decision-making. However, in a subset of cases, it may remain inconclusive, particularly in the hyperacute phase, where perfusion and diffusion changes are dynamic and potentially non-specific. Table 1 summarizes the main imaging patterns and clinical pitfalls in the differential diagnosis between acute ischemic stroke and seizure across various CT and MRI modalities. The most reliable discriminator is vascular-territorial congruence: ischemic stroke typically produces abnormalities that follow a specific arterial distribution supported by angiographic evidence of occlusion or stenosis, whereas seizure-related changes are primarily cortical, non-territorial, and dynamically linked to the ictal or post-ictal phase [28,50].

From a practical stroke-triage perspective, this distinction can be framed within the “3C’s” and “4P’s” frameworks, which integrate infarct core, arterial clot or occlusion, collateral status and tissue perfusion/penumbra (parenchyma, pipes, perfusion, and penumbra) [56,57,58]. These models provide a structured way to assess whether imaging findings are internally consistent with an ischemic mechanism or instead suggest a seizure-related process, helping to interpret equivocal perfusion findings. In this context, EEG should be considered early when clinical–imaging findings are discordant with a vascular-territorial mechanism, particularly in cases of unexplained or fluctuating altered consciousness, persistent focal deficits, isolated aphasia without clear arterial occlusion, or imaging patterns suggestive of seizure activity, including non-territorial cortical perfusion abnormalities, while remaining a complementary tool that should not delay urgent reperfusion therapy when acute ischemic stroke is strongly supported [6,58,59]. The most common EEG patterns observed in the acute phase of stroke include focal slowing, lateralized periodic discharges, sporadic focal epileptiform abnormalities, generalized rhythmic delta activity and attenuation of background activity in the affected hemisphere. Quantitative EEG parameters, particularly asymmetry metrics and the alpha–delta ratio, offer valuable clinical utility in detecting evolving tissue ischemia and refining the prognostication of long-term functional outcomes [60]. Moreover, prolonged and continuous video-EEG may be useful for detecting non-convulsive status epilepticus, particularly in patients with fluctuating consciousness, unexplained focal deficits or subtle recurrent motor phenomena, especially when clinical and imaging findings are discordant [61,62].

In acute CT-first pathways, CT perfusion is highly valuable but demands cautious interpretation. While territorial hypoperfusion concordant with CTA strongly supports ischemia, the ictal or peri-ictal phases of a seizure often manifest as cortical hyperperfusion. Furthermore, post-ictal states or Todd’s phenomena may exhibit normal perfusion or hypoperfusion that closely mimics an ischemic penumbra. Consequently, the direction of perfusion change alone is insufficient for a definitive diagnosis; it must be interpreted in the context of spatial distribution, vascular anatomy, and clinical timing. MRI provides critical complementary data to navigate these overlaps. Although DWI and ADC maps remain central for detecting early ischemia, reversible diffusion restriction can also occur in peri-ictal states. To aid in differentiation, ASL supplies non-contrast perfusion information that can strongly support an epileptic mechanism (particularly in suspected SE) when it reveals cortical or seizure-network-related hyperperfusion [53,55]. However, like CTP, ASL is also vulnerable to diagnostic pitfalls, and its interpretation must carefully account for transit-delay artifacts, reperfusion-related “luxury” hyperperfusion, and post-ictal hypoperfusion [13]. Importantly, the generalizability of CTP and ASL findings is constrained by substantial technical and clinical heterogeneity, including variations in acquisition protocols, post-processing software, perfusion thresholds, transit delays, and timing from symptoms or seizure onset. Furthermore, a widespread lack of predefined selection criteria and formal quality assessments introduces a significant risk of selection bias across literature. Ultimately, the persistence of these diagnostic uncertainties highlights the need for more integrated and standardized research protocols.

Functional nuclear imaging, including ictal SPECT (detecting regional hyperperfusion) and interictal FDG-PET (showing regional hypometabolism), can provide valuable complementary insights in highly selected, ambiguous stroke-mimic cases [63,64], such as aphasic status epilepticus [65]. However, given their limited availability, complex radiotracer logistics, and prolonged workflows, these modalities are extremely difficult to perform in the hyperacute phase. Consequently, they do not represent first-line emergency triage tools and must be reserved as specialized diagnostic adjuncts for unresolved or subacute cases.

Future research should move beyond isolated descriptions of imaging patterns and directly compare CTP, DWI/ADC, FLAIR, and ASL within the same hyperacute stroke-code cohorts. A key priority is the standardization of imaging timing, given the highly dynamic nature of seizure-related perfusion abnormalities, which may evolve from ictal hyperperfusion to post-ictal hypoperfusion over short time intervals. Prospective studies should adopt EEG-confirmed seizure or SE as the reference standard and specifically address the diagnostic uncertainty of Todd’s phenomena, where focal deficits and hypoperfusion may closely mimic ischemic penumbra. In addition, quantitative thresholds for CTP and ASL parameters, including severity, distribution, and territoriality of perfusion changes, should be systematically validated against clinical outcomes and follow-up imaging. Artificial intelligence may further refine this diagnostic framework by integrating multimodal imaging with clinical and temporal variables. Machine-learning approaches applied to CTA/CTP, DWI/ADC, FLAIR, ASL, EEG data and clinical metadata could help identify subtle and complex patterns that are difficult to recognize visually, such as non-territorial cortical perfusion abnormalities, early peri-ictal diffusion changes, or post-ictal hypoperfusion mimicking ischemic penumbra [66]. AI-assisted approaches, including machine-learning perfusion analysis, automated LVO detection and automated collateral scoring, can accelerate triage, reduce workflow delays and improve diagnostic consistency [67,68]. By integrating multimodal data (CTA/CTP, MRI, EEG, and metadata), these models can detect subtle patterns difficult to recognize visually [69]. However, in the stroke–seizure differential, AI outputs must be interpreted cautiously. Seizure-related hyperperfusion, post-ictal hypoperfusion, and motion artifacts can confound automated classification, risking false positives or negatives. Therefore, AI should serve as a decision-support tool rather than an autonomous diagnostic tool, and its clinical translation will require robust external validation across diverse workflows using large, prospectively collected, EEG-annotated datasets enriched for stroke mimics before implementation in routine practice.

## 5. Conclusions

Advanced imaging can substantially refine the differential diagnosis between acute ischemic stroke and seizure-related deficits, yet no single modality is definitive in isolation. The most robust approach relies on a multimodal integration of CTA/CTP, DWI/ADC/FLAIR, and ASL interpreted alongside clinical presentation, symptom evolution, and, when available, EEG findings. EEG should be considered early when clinical and imaging findings are discordant with a vascular-territorial mechanism, focal deficits persist despite absent arterial occlusion, or non-convulsive status epilepticus is suspected, while remaining complementary to vascular and perfusion imaging and not delaying reperfusion therapy in acute ischemic stroke. Ultimately, accurate diagnosis depends on integrating imaging and other diagnostic findings within their clinical and temporal context rather than relying on any single parameter.

## Figures and Tables

**Table 1:** Main imaging patterns and clinical pitfalls in differential diagnosis between acute ischemic stroke and seizure.

Imaging Modality	Stroke Pattern	Seizure/Peri-Ictal Pattern	Main Pitfalls/Limitations
CT: NCCT	Early ischemic changes (loss of gray–white differentiation, insular ribbon sign, sulcal effacement, hyperdense vessel) typically within a vascular territory. Often normal in hyperacute phase.	Usually normal; occasionally transient cortical hypodensity or swelling, often non-territorial	Low sensitivity in early stroke; seizure-related changes may overlap with early ischemia
CTA	Frequent LVO (ICA, M1, basilar) with anatomically congruent deficit	Typically, no occlusion; normal or mildly altered vessel caliber	Distal occlusions, early recanalization or chronic stenosis may confound interpretation
CTP	Territorial hypoperfusion pattern: core (CBF↓, CBV↓) + penumbra (Tmax↑, MTT↑)	Cortical, non-territorial, dynamic changes: - ictal: hyperperfusion (CBF↑, CBV↑, MTT↓); - post-ictal: variable hypo-perfusion	Post-ictal hypoperfusion may mimic stroke; findings highly time-dependent
MRI: DWI	Persistent restriction, with low ADC, usually territorial	Negative or transient, reversible restriction (cortex, hippocampus), non-territorial	Early stroke may be DWI-negative; seizure-related restriction may mimic infarction
FLAIR	Delayed changes (over hours), typically negative early; DWI–FLAIR mismatch indicates hyperacute stroke	Often normal or mild cortical hyperintensity; inconsistent temporal evolution	Overlap in subacute phase; prolonged seizures or status epilepticus may show FLAIR abnormalities
ASL	Hypoperfusion in a vascular territory, often with delayed transit effects	Focal, non-territorial, network-based changes: - ictal: hyperperfusion (cortical ± thalamic/pulvinar) - post-ictal: hypoperfusion	Transit delay artifacts; post-ictal hypoperfusion may resemble ischemia

ADC: Apparent Diffusion Coefficient; ASL: Arterial Spin Labeling; CBF: Cerebral Blood Flow; CBV: Cerebral Blood Volume; CT: Computed Tomography; CTA: Computed Tomography Angiography; CTP: Computed Tomography Perfusion; DWI: Diffusion-Weighted Imaging; FLAIR: Fluid-Attenuated Inversion Recovery; ICA: Internal Carotid Artery; LVO: Large Vessel Occlusion; MTT: Mean Transit Time; MRI: Magnetic Resonance Imaging; NCCT: Non-Contrast Computed Tomography.

## Data Availability

No new data were created or analyzed in this study. Data sharing is not applicable to this article.

## Abbreviations

The following abbreviations are used in this manuscript:

ADC	Apparent Diffusion Coefficient
AIS	Acute Ischemic Stroke
ASL	Arterial Spin Labeling
CBF	Cerebral Blood Flow
CBV	Cerebral Blood Volume
CT	Computed Tomography
CTA	Computed Tomography Angiography
CTP	Computed Tomography Perfusion
DSC	Dynamic Susceptibility Contrast
DWI	Diffusion-Weighted Imaging
DWIR	Diffusion-Weighted Imaging Reversal
EEG	Electroencephalogram
FLAIR	Fluid-Attenuated Inversion Recovery
ICA	Internal Carotid Artery
LVO	Large Vessel Occlusion
MCA	Middle Cerebral Artery
MRI	Magnetic Resonance Imaging
MTT	Mean Transit Time
NCCT	Non-Contrast Computed Tomography
NIHSS	National Institutes of Health Stroke Scale
PWI	Perfusion-Weighted Imaging
SE	Status Epilepticus

## References

[1] Buck B.H., Akhtar N., Alrohimi A., Khan K., Shuaib A. (2021). Stroke mimics: Incidence, aetiology, clinical features and treatment. Ann. Med..

[2] Khawar H., McFarlane S., Libman R.B. (2025). Stroke Mimics at 30 Years: Where We Have Been, Where We Are Now, and Where We Are Going. Stroke.

[3] Pohl M., Hesszenberger D., Kapus K., Meszaros J., Feher A., Varadi I., Pusch G., Fejes E., Tibold A., Feher G. (2021). Ischemic stroke mimics: A comprehensive review. J. Clin. Neurosci..

[4] Vilela P. (2017). Acute stroke differential diagnosis: Stroke mimics. Eur. J. Radiol..

[5] Wenstrup J., Hestoy B.H., Sagar M.V., Blomberg S.N.F., Christensen H., Christensen H.C., Kruuse C. (2024). Emergency Medical Services dispatcher recognition of stroke: A systematic review. Eur. Stroke J..

[6] Brigo F., Lattanzi S. (2020). Poststroke seizures as stroke mimics: Clinical assessment and management. Epilepsy Behav..

[7] Prabhakaran S., Gonzalez N.R., Zachrison K.S., Adeoye O., Alexandrov A.W., Ansari S.A., Chapman S., Czap A.L., Dumitrascu O.M., Ishida K. (2026). 2026 Guideline for the Early Management of Patients with Acute Ischemic Stroke: A Guideline from the American Heart Association/American Stroke Association. Stroke.

[8] Zinkstok S.M., Engelter S.T., Gensicke H., Lyrer P.A., Ringleb P.A., Artto V., Putaala J., Haapaniemi E., Tatlisumak T., Chen Y. (2013). Safety of thrombolysis in stroke mimics: Results from a multicenter cohort study. Stroke.

[9] Pereira A., Alakbarzade V., Lowe D., Hargroves D. (2025). Computed Tomography Perfusion in Acute Stroke Assessment. Cerebrovasc. Dis..

[10] Tedyanto E.H., Tini K., Pramana N.A.K. (2022). Magnetic Resonance Imaging in Acute Ischemic Stroke. Cureus.

[11] Rapai Kasini N.A., Payus A.O., Khoo C.S., Tan H.J., Mohamed Mukari S.A., Mohd Mustapha A.M., Hod R. (2025). Determining the computed tomography perfusion changes in patients presenting with acute seizures of different etiologies—A case-control study. Epilepsy Behav..

[12] Pascarella A., Manzo L., Marsico O., Africa E., Coglitore A., Cianci V., Bulgari A., Abelardo D., Gasparini S., Armentano A. (2025). Investigating Peri-Ictal MRI Abnormalities: A Prospective Neuroimaging Study on Status Epilepticus, Seizure Clusters, and Single Seizures. J. Clin. Med..

[13] Schertz M., Benzakoun J., Pyatigorskaya N., Belkacem S., Sahli-Amor M., Navarro V., Cholet C., Leclercq D., Dormont D., Law-Ye B. (2020). Specificities of arterial spin labeling (ASL) abnormalities in acute seizure. J. Neuroradiol..

[14] Barber P.A., Demchuk A.M., Zhang J., Buchan A.M. (2000). Validity and reliability of a quantitative computed tomography score in predicting outcome of hyperacute stroke before thrombolytic therapy. ASPECTS Study Group. Alberta Stroke Programme Early CT Score. Lancet.

[15] Kucinski T. (2005). Unenhanced CT and acute stroke physiology. Neuroimaging Clin. N. Am..

[16] Palmeri B., Talamanca S., Ragonese P., Aridon P., Daniele O., Terruso V., D’Amelio M. (2011). MRI abnormalities following repeated and incoming seizures. Seizure.

[17] Cole A.J. (2004). Status epilepticus and periictal imaging. Epilepsia.

[18] Powers W.J., Rabinstein A.A., Ackerson T., Adeoye O.M., Bambakidis N.C., Becker K., Biller J., Brown M., Demaerschalk B.M., Hoh B. (2019). Guidelines for the Early Management of Patients with Acute Ischemic Stroke: 2019 Update to the 2018 Guidelines for the Early Management of Acute Ischemic Stroke: A Guideline for Healthcare Professionals from the American Heart Association/American Stroke Association. Stroke.

[19] Hand P.J., Kwan J., Lindley R.I., Dennis M.S., Wardlaw J.M. (2006). Distinguishing between stroke and mimic at the bedside: The brain attack study. Stroke.

[20] Tobin W.O., Hentz J.G., Bobrow B.J., Demaerschalk B.M. (2009). Identification of stroke mimics in the emergency department setting. J. Brain Dis..

[21] Fasen B., Heijboer R.J.J., Hulsmans F.H., Kwee R.M. (2021). Diagnostic performance of single-phase CT angiography in detecting large vessel occlusion in ischemic stroke: A systematic review. Eur. J. Radiol..

[22] Konstas A.A., Goldmakher G.V., Lee T.Y., Lev M.H. (2009). Theoretic basis and technical implementations of CT perfusion in acute ischemic stroke, part 1: Theoretic basis. AJNR Am. J. Neuroradiol..

[23] Nicolas-Jilwan M., Wintermark M. (2021). Automated Brain Perfusion Imaging in Acute Ischemic Stroke: Interpretation Pearls and Pitfalls. Stroke.

[24] Kargiotis O., Psychogios K., Safouris A., Andrikopoulou A., Eleftheriou A., Spiliopoulos S., Magoufis G., Tsivgoulis G. (2023). Computed Tomography Perfusion Imaging in Acute Ischemic Stroke: Accurate Interpretation Matters. Stroke.

[25] Campbell B.C., Weir L., Desmond P.M., Tu H.T., Hand P.J., Yan B., Donnan G.A., Parsons M.W., Davis S.M. (2013). CT perfusion improves diagnostic accuracy and confidence in acute ischaemic stroke. J. Neurol. Neurosurg. Psychiatry.

[26] Campbell B.C., Mitchell P.J., Kleinig T.J., Dewey H.M., Churilov L., Yassi N., Yan B., Dowling R.J., Parsons M.W., Oxley T.J. (2015). Endovascular therapy for ischemic stroke with perfusion-imaging selection. N. Engl. J. Med..

[27] Ma H., Campbell B.C.V., Parsons M.W., Churilov L., Levi C.R., Hsu C., Kleinig T.J., Wijeratne T., Curtze S., Dewey H.M. (2019). Thrombolysis Guided by Perfusion Imaging up to 9 Hours after Onset of Stroke. N. Engl. J. Med..

[28] Austein F., Huhndorf M., Meyne J., Laufs H., Jansen O., Lindner T. (2018). Advanced CT for diagnosis of seizure-related stroke mimics. Eur. Radiol..

[29] Gugger J.J., Llinas R.H., Kaplan P.W. (2020). The role of CT perfusion in the evaluation of seizures, the post-ictal state, and status epilepticus. Epilepsy Res..

[30] Lucas L., Gariel F., Menegon P., Aupy J., Thomas B., Tourdias T., Sibon I., Renou P. (2021). Acute Ischemic Stroke or Epileptic Seizure? Yield of CT Perfusion in a “Code Stroke” Situation. AJNR Am. J. Neuroradiol..

[31] Strambo D., Rey V., Rossetti A.O., Maeder P., Dunet V., Browaeys P., Michel P. (2018). Perfusion-CT imaging in epileptic seizures. J. Neurol..

[32] Restrepo-Vera J.L., Coscojuela P., Fonseca E., Quintana M., Sarria-Estrada S., Santamarina E., Abraira L., Sueiras M., Thonon V., Álvarez-Sabin J. (2022). Epileptic seizures in the emergency room: Clinical and electroencephalographic findings associated with brain perfusion patterns on computed tomography. J. Neurol..

[33] Giovannini G., Malagoli M., Turchi G., Miani A., Orlandi N., Vaudano A.E., Meletti S. (2021). Cortical and thalamic hyper-perfusion in non-convulsive status epilepticus. Relationship between perfusion CT patterns and Salzburg EEG criteria. Seizure.

[34] Rudilosso S., Rodríguez A., Amaro S., Obach V., Renú A., Llull L., Laredo C., Marín Á.A., Vargas A., Urra X. (2020). Value of Vascular and Non-Vascular Pattern on Computed Tomography Perfusion in Patients with Acute Isolated Aphasia. Stroke.

[35] Prodi E., Danieli L., Manno C., Pagnamenta A., Pravatà E., Roccatagliata L., Städler C., Cereda C.W., Cianfoni A. (2022). Stroke Mimics in the Acute Setting: Role of Multimodal CT Protocol. AJNR Am. J. Neuroradiol..

[36] Nagaraja N. (2021). Diffusion weighted imaging in acute ischemic stroke: A review of its interpretation pitfalls and advanced diffusion imaging application. J. Neurol. Sci..

[37] Nukovic J.J., Opancina V., Ciceri E., Muto M., Zdravkovic N., Altin A., Altaysoy P., Kastelic R., Velazquez Mendivil D.M., Nukovic J.A. (2023). Neuroimaging Modalities Used for Ischemic Stroke Diagnosis and Monitoring. Medicina.

[38] Thomalla G., Simonsen C.Z., Boutitie F., Andersen G., Berthezene Y., Cheng B., Cheripelli B., Cho T.H., Fazekas F., Fiehler J. (2018). MRI-Guided Thrombolysis for Stroke with Unknown Time of Onset. N. Engl. J. Med..

[39] Adam G., Ferrier M., Patsoura S., Gramada R., Meluchova Z., Cazzola V., Darcourt J., Cognard C., Viguier A., Bonneville F. (2018). Magnetic resonance imaging of arterial stroke mimics: A pictorial review. Insights Imaging.

[40] Finelli P.F., Nouh A. (2016). Three-Territory DWI Acute Infarcts: Diagnostic Value in Cancer-Associated Hypercoagulation Stroke (Trousseau Syndrome). AJNR Am. J. Neuroradiol..

[41] Fernandes D.A., Santos F.B.A., Pereira F.V., Teixeira C.E.G., Sachetto Z., Boin I., Carvalheira J.B.C., Reis F. (2025). Trousseau syndrome preceding the diagnosis of colon cancer. Rev. Esp. Enferm. Dig..

[42] Kim K., Kim B.J., Huh J., Yang S.K., Yang M.H., Han M.K., Jung C., Choi B.S., Kim J.H., Bae H.J. (2021). Delayed Lesions on Diffusion-Weighted Imaging in Initially Lesion-Negative Stroke Patients. J. Stroke.

[43] Edlow B.L., Hurwitz S., Edlow J.A. (2017). Diagnosis of DWI-negative acute ischemic stroke: A meta-analysis. Neurology.

[44] Makin S.D., Doubal F.N., Dennis M.S., Wardlaw J.M. (2015). Clinically Confirmed Stroke with Negative Diffusion-Weighted Imaging Magnetic Resonance Imaging: Longitudinal Study of Clinical Outcomes, Stroke Recurrence, and Systematic Review. Stroke.

[45] Williams J., Mullins G., Delanty N., Bede P., Doherty C.P. (2017). The spectrum of peri-ictal MRI changes; four illustrative cases. Seizure.

[46] Hübers A., Thoma K., Schocke M., Fauser S., Ludolph A.C., Kassubek J., Pinkhardt E.H. (2018). Acute DWI Reductions In Patients After Single Epileptic Seizures—More Common Than Assumed. Front. Neurol..

[47] Dang H.Q., Nguyen T.Q., Chiem D.N., Le T.V.S., Nguyen R.A., Nguyen H., Nguyen T.H. (2024). Near-Complete Reversal of Large Diffusion-Weighted Imaging Lesion after Thrombectomy: A Case Report and Literature Review. Cerebrovasc. Dis. Extra.

[48] Huang T.H., Lai M.C., Chen Y.S., Huang C.W. (2022). Brain Imaging in Epilepsy-Focus on Diffusion-Weighted Imaging. Diagnostics.

[49] Buch K., Hakimelahi R., Locascio J.J., Bolar D.S., Gonzalez R.G., Schaefer P.W. (2022). Clinical utility of arterial spin labeling perfusion images in the emergency department for the work-up of stroke-like symptoms. Neuroradiology.

[50] Zaharchuk G. (2014). Arterial spin-labeled perfusion imaging in acute ischemic stroke. Stroke.

[51] Daftari Besheli L., Ahmed A., Hamam O., Luna L., Sun L.R., Urrutia V., Hillis A.E., Tekes-Brady A., Yedavalli V. (2022). Arterial Spin Labeling technique and clinical applications of the intracranial compartment in stroke and stroke mimics—A case-based review. Neuroradiol. J..

[52] Sato T., Katagiri M., Inoue T., Kubo S., Sato K., Himeno T., Itaya T., Terasawa Y. (2026). Arterial spin labeling imaging of epileptic seizures with and without status epilepticus: A descriptive study. Acta Neurol. Belg..

[53] Bosque Varela P., Machegger L., Prüwasser T., Lauth W., Steinbacher J., Oellerer A., Zimmermann G., Pfaff J., McCoy M., Crespo Pimentel B. (2026). Timing of Peri-Ictal MRI Abnormalities in Status Epilepticus—One Size Does Not Fit All. Ann. Neurol..

[54] Kim T.J., Choi J.W., Han M., Kim B.G., Park S.A., Huh K., Choi J.Y. (2021). Usefulness of arterial spin labeling perfusion as an initial evaluation of status epilepticus. Sci. Rep..

[55] Bosque-Varela P., Kuchukhidze G., Hahn W., Jünemann C., Machegger L., Möller L., Pfaff J., Trinka E., Knake S., Tsalouchidou P.E. (2025). Peri-ictal imaging abnormalities in non-convulsive status epilepticus: A systematic review and meta-analysis comparing magnetic resonance imaging and computed tomography perfusion. Epilepsia.

[56] Rowley H.A. (2001). The four Ps of acute stroke imaging: Parenchyma, pipes, perfusion, and penumbra. AJNR Am. J. Neuroradiol..

[57] Zhao H., Pesavento L., Coote S., Rodrigues E., Salvaris P., Smith K., Bernard S., Stephenson M., Churilov L., Yassi N. (2018). Ambulance Clinical Triage for Acute Stroke Treatment: Paramedic Triage Algorithm for Large Vessel Occlusion. Stroke.

[58] Kim J.W., Stetefeld H.R., Fink G.R., Malter M.P. (2023). Seizures at stroke onset: A case-control study. Seizure.

[59] Guerrero W.R., Dababneh H., Eisenschenk S. (2012). The role of perfusion CT in identifying stroke mimics in the emergency room: A case of status epilepticus presenting with perfusion CT alterations. Int. J. Emerg. Med..

[60] Leclercq V., Gaspard N. (2026). Electroencephalography in stroke Care: From acute monitoring to Post-Stroke epilepsy. Clin. Neurophysiol. Pract..

[61] Bitar R., Khan U.M., Rosenthal E.S. (2024). Utility and rationale for continuous EEG monitoring: A primer for the general intensivist. Crit. Care.

[62] Belcastro V., Vidale S., Gorgone G., Pisani L.R., Sironi L., Arnaboldi M., Pisani F. (2014). Non-convulsive status epilepticus after ischemic stroke: A hospital-based stroke cohort study. J. Neurol..

[63] Kim S., Mountz J.M. (2011). SPECT Imaging of Epilepsy: An Overview and Comparison with F-18 FDG PET. Int. J. Mol. Imaging.

[64] Sarikaya I. (2015). PET studies in epilepsy. Am. J. Nucl. Med. Mol. Imaging.

[65] Manganotti P., Furlanis G., Cova M.A., Olivo S., Dore F., Sartori A., Naccarato M. (2022). Isolated aphasic status epilepticus: CT perfusion, SPECT and EEG reveal neurovascular coupling and support the differential diagnosis. Epileptic Disord..

[66] Heo J. (2024). Application of Artificial Intelligence in Acute Ischemic Stroke: A Scoping Review. Neurointervention.

[67] Martinez-Gutierrez J.C., Kim Y., Salazar-Marioni S., Tariq M.B., Abdelkhaleq R., Niktabe A., Ballekere A.N., Iyyangar A.S., Le M., Azeem H. (2023). Automated Large Vessel Occlusion Detection Software and Thrombectomy Treatment Times: A Cluster Randomized Clinical Trial. JAMA Neurol..

[68] Bagcilar O., Alis D., Alis C., Seker M.E., Yergin M., Ustundag A., Hikmet E., Tezcan A., Polat G., Akkus A.T. (2023). Automated LVO detection and collateral scoring on CTA using a 3D self-configuring object detection network: A multi-center study. Sci. Rep..

[69] Kim J.G., Ha S.Y., Kang Y.R., Hong H., Kim D., Lee M., Sunwoo L., Ryu W.S., Kim J.T. (2025). Automated detection of large vessel occlusion using deep learning: A pivotal multicenter study and reader performance study. J. NeuroInterv. Surg..

